# Influence of nano silicon dioxide from sand and rice straw ash on broiler productivity, antioxidant status and bone quality

**DOI:** 10.1038/s41598-025-07972-w

**Published:** 2025-07-07

**Authors:** Ahmed Samy, Hany M. R. Elsherif

**Affiliations:** 1 Department of Animal Production, National Research Centre, Cairo, 12622 Egypt; 2 Animal Production Department, Faculty of Agriculture, Cairo University, Giza, Egypt

**Keywords:** Antioxidants status, Biochemical blood, Broiler performance, Nano silicon oxide, Tibia bone characteristics, Thyroid hormones, Animal physiology, Nanoparticles

## Abstract

This study evaluated the effects of sand- and rice straw ash-derived nanosilicon dioxide (NSis and NSia) on broiler chickens’ performance, blood biochemistry, antioxidant status, and tibia bone characteristics. Five hundred forty 10-day-old Arbor Acres male chicks were divided into nine groups, each with six replicates of ten chicks. Diets included a control group without additives, while others were supplemented with sand, rice straw ash, or their nanosilicon derivatives (NSis and NSia) at 300 ppm and 600 ppm. Results indicated significant improvements (*P* ≤ 0.05) in weight gain and feed conversion ratio in all treatments, with the best outcomes observed in chicks fed diets enriched with NSis or NSia. Blood parameters showed enhanced globulin levels and albumin/globulin ratios. Antioxidant status, assessed by total antioxidant capacity, catalase, and malondialdehyde levels, improved significantly (*P* ≤ 0.05) with NSis or NSia. Tibia bone characteristics, including width, breaking strength, and bone mineral density and concentration, were also significantly enhanced. No adverse effects on kidney or liver functions or thyroid hormones were observed across all treatments. These findings suggest that enriching broiler diets with sand or rice straw ash-derived nanosilicon dioxide can improve productivity, bone quality, and antioxidant status without any harmful effects.

## Introduction

Silicon is an essential trace mineral in animal diets because it’s usefulness in maintaining healthy connective tissue, normal development of bone and enhanced the quality bone^1–3^. Genetic selection and/or improved feeding efficiency have led to an increase in the production rate of animal protein sources like broilers, which has resulted in an earlier slaughter weight, increasing the risk of their skeletal problems^4–6^. Lu et al.^7^ have revealed the synergistic effects of calcium and silicon for the enhancement of bone growth. Thus, calcium metabolism and the stability of the extracellular bone matrix appear to be related to silicon^8^. In addition to, bone calcification and decalcification processes can be influenced due to the regulation of calcium turnover by silicon^9^. Consequently, limiting the amount of Si in animals’ diets while they were growing led to irregularities in growth, connective tissue, and bone diseases^10^. Prentice^1^ revealed that modern broiler chickens do not appear to acquire enough bio-available silicon in their conventional diets for optimal skeletal growth, and that bioavailable silicon supplementation would benefit them.

**Fig. 1:**
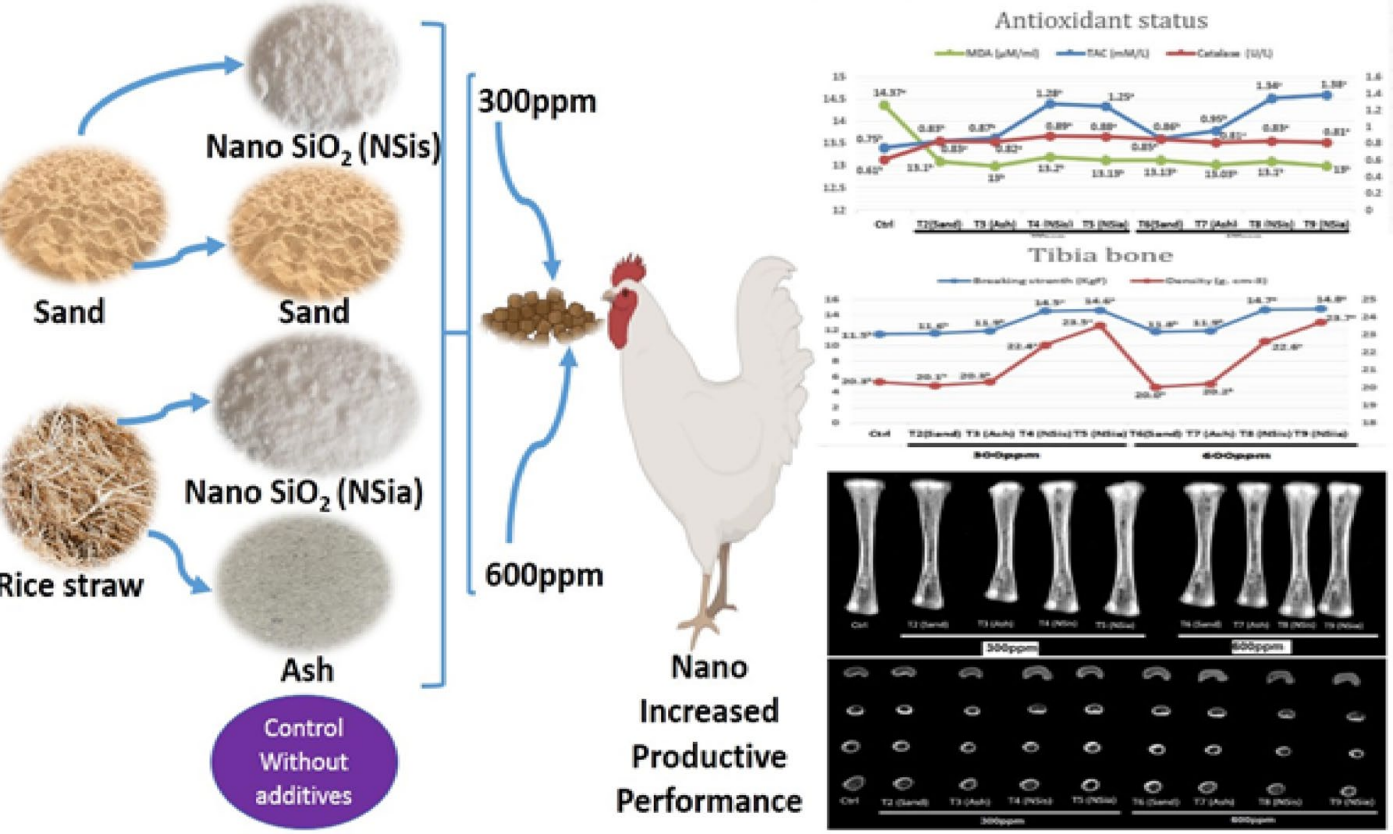
Graphical abstract.

Many factors influence the trace mineral’s bioavailability. The bioavailability of trace minerals associated with bone health has been improved by the use of nanotechnology in mineral development^11^. Nanominerals have unique characteristics due to their very small size between 1 and 100 nm whish give them the superior effect over the conventional form, whereas increasing the activity, reactivity, absorption and utilization so maximize their bioavailability^6,12–14^. Protection from chemical conditions in the gastrointestinal tract and better transfer across the intestinal wall are two ways in which nanominerals boost their bioavailability^15,16^. Particle size, the physical condition of nanominerals, and their surface characteristics all affect the nutritional value of nanominerals^17^. Modern broiler strains may necessitate the use of bioavailable silicon supplements in their feed. Burton et al.^18^ found that adding silica (1000 ppm) to the broiler diet increased weight gain and bone strength without changing bone mineral content. Furthermore, this study was conducted to investigate the influence of the nano silicon dioxide produced from sand and rice straw’s ash at two different levels (300 and 600 ppm) on the broiler productive performance, antioxidants status, blood biochemical and tibia bone characteristics during the experimental period (11–33 days of age) as shown in Fig. 1, the graphical abstract.

## Materials and methods

### Ethical approval and animal welfare statement

The animal trial was approved by the National Research Center Medical Research Ethics Committee (Approval Number: 13050405). The study complied with the Helsinki Declaration, Good Medical and Laboratory Practice, Institutional Animal Care and Use Committee (IACUC) guidelines, International Health Organization regulations, and applicable Egyptian laws. Animal care and use followed European Union (EU) guidelines, including compliance with the ARRIVE guidelines (https://arriveguidelines.org/).

### Anesthesia and Euthanasia Statement

All procedures involving animals were conducted in accordance with veterinary best practices. Anesthesia was induced using a combination of ketamine (20–40 mg/kg) and xylazine (2–4 mg/kg) to ensure adequate sedation and pain relief. Euthanasia was performed by 80% CO₂ inhalation, gradually introduced into the chamber at a displacement rate of approximately 20–30% of the chamber volume per minute, in accordance with the American Veterinary Medical Association (AVMA) Guidelines for the Euthanasia of Animals (2020). This method ensures a humane and painless death by inducing hypoxia, leading to unconsciousness and subsequent euthanasia.

### Birds, housing and management

Experiments were carried out on the farm at the Poultry Nutrition Research Unit (PNRU), Cairo University’s faculty of Agriculture in Giza, Egypt while, the synthesis of nanomaterials and experimental analyses at the National Research Centre’s laboratory for animal production.

### Materials

The dietary sources of silicon dioxide used in this study, sand from one of the Egyptian stores, rice straw from the remnants of rice farms^19^. The rice straw was collected, thoroughly washed with distilled water to remove dirt and impurities, air-dried, and then incinerated in a muffle furnace at 900 °C for 6 h to obtain rice straw ash. The Nano silicon dioxide produced from sand or rice straw’s ash were synthesized using sol–gel method at pH 8 in laboratory of animal production Department, Biological and agricultural Insinuate, National Research Centre, Egypt^20,21^.

#### Animals and experiment design

In three-layer batteries, four different silicon sources were compared in a broiler chick’s growth trial for 11–33 days of age, with two levels of each. Five hundred and forty Arbor acres broiler male chicks weighing around 310 g at 10 days of age were randomly assigned into nine groups, each with six replicates of ten chicks each. Nine experimental diets were formulated, the control group containing no additives, while diets 2, 3, 4, and 5 were supplemented with sand, rice straw’s ash, nano silicon dioxide produced from sand and nano silicon dioxide produced from rice straw’s ash, respectively, at 300 ppm. Diets 6–9 contained the highest level of diets 2–5, respectively, at 600 ppm. The diets’ formulation and nutrient composition are shown in Table 1. Throughout the study, feed and water were always available; a vaccination protocol against IB, IBD, avian flu and New Castle was meticulously followed.

**Table 1:** Formulation and nutrients composition of the growr and finisher diets.

Ingredients %	Period	
	Grower (11–25 days)	Finisher (26–33 days)
Yellow maize	56.03	63.00
46% Soybean meal	31.20	25.13
Gluten meal (60%)	5.30	5.00
Soybean oil	3.50	3.10
Limestone	1.04	0.93
Mono calcium phosphate	1.60	1.58
Common salt (NaCl)	0.20	0.20
NaHCO_3_	0.23	0.23
Vitamin and mineral mix^a^	0.30	0.30
DL-methionine	0.14	0.13
L-lysine HCl	0.32	0.28
Threonine	0.09	0.07
Choline chloride	0.05	0.05
Total	**100**	**100**
Calculated composition^b^		
Crude protein %	21.50	20.0
ME (Kcal/kg)	3100	3200
Ether extract%	6.01	5.87
Crude fiber%	3.49	2.43
Lysine %	1.29	1.19
Methionine %	0.51	0.51
Methionine + Cystine %	0.88	0.87
Threonine %	0.88	0.81
Calcium %	0.87	0.81
Nonphytate P %	0.435	0.405
Sodium %	0.16	0.16
Chlorine %	0.16	0.16

^a^Per kg of diet, the following vitamin-mineral mixture is provided: Vitamin A, 12,000 IU; Vitamin E, 10 mg; Vitamin D3, 2200 IU; Vitamin K3, 2 mg; Vitamin B1, 1 mg; Vitamin B6, Vit B2, 4 mg; 1.5 mg; Vitamin B12, 10 mg; Pantothenic acid, 10 mg; Niacin, 20 mg; Folic acid, 1 mg; Biotin, 50 mg; Choline chloride, 500 mg; Iodine, 1 mg; Copper, 10 mg; Iron, 30 mg; Selenium, 0.1 mg; Zinc, 50 mg and Manganese, 55 mg.

^b^According to NRC 1994.

#### Growth performance

The birds were weighed and their feed consumption was recorded per replicate at 26 and 33 days of age in order to assess weight gain and feed conversion ratio^6–21^.

#### Collection of blood serum samples

After a 6-h fast, blood was obtained from 5 birds per treatment at the slaughter at 33 days of age. The samples were identified and centrifuged for ten minutes at 2000 rpm. The serum collected was kept in a freezer until analyze it later^22^.

#### Analysis of serum for

##### Blood proteins

Total protein, albumin, and calculated globulin by subtracting albumin from total protein, as well as the albumin globulin ratio (albumin divided by globulin).

##### Liver and kidney function

Alkaline phosphatase (ALP), Urea, alanine aminotransferase (ALT) and aspartate aminotransferase (AST).

##### Antioxidants status

TAC (total antioxidant capacity), CAT (catalase), and MDA (malondialdehyde).

All biochemical blood parameters were measured using spectrophotometer (FlexorEL200 Biochemical Analyzer) using commercial kits (biodiagnostic, Cairo, Egypt).

##### Thyroid hormone analysis

Using commercial ELISA kits (MyBioSource, Inc., San Diego, CA), the total T3 and T4 serum concentrations were determined. Thus, by dividing the value of T3 by the value of T4, the T3/T4 ratio was computed.

#### Characteristics of tibia bone

At 33d, the right tibia bone was removed and prepped for the various measurements. Tibia was washed of all adherent flesh, extracted with ethanol, and then extracted with diethyl ether. After measuring the weight, length, and width of the tibia, the bones were oven dried at 105 °C for constant weight. The Digital Force Gauge equipment was used to determine the tibia’s breaking strength, which was expressed in kilogrammes of force required to break the bone^23^. Dry fat-free tibia samples were ashed in a muffle furnace at 600°C for 6 h.

#### X-ray of the tibia bone

The effects of various treatments on X-ray scans of tibia bone mineral density were investigated using radiographic images of the tibia^5^.

#### Tibia bone computerized tomography (CT)

A GE Optima CT660 CT Scanner is used to make cross-sectional photographs (slices) of the bones by combining a series of X-ray images taken from various angles for tibia bone mineral density measurements (BMD).

#### Statistical analysis

Statistical analyses were performed using SAS software. The normality of data distribution was assessed using the Shapiro–Wilk test, and the results confirmed that the data were normally distributed (*p* > 0.05). Based on this, SAS’s General Liner Model (one-way and two-way analysis of variance) were used to examine the data^24^. The differences between the treatment means that were separated using Duncan’s new multiple range test at (*P* ≤ 0.05) were significant^25^.

## Results

### Growth performance

Table 2 shows the impact of several nutritional treatments on the grower (11–25 d), finisher (26–33 d), and overall periods (11–33 d).

**Table 2:** The impact of several nutritional treatments on the grower (11-25d), finisher (26-33d), and overall periods (11-33d).

Item		Start weight (g, 10d)	Grower period (11–25)			Finisher period (26–33)			Overall period (11–33)		
			WG(g)	FI (g)	FCR	WG(g)	FI (g)	FCR	WG(g)	FI (g)	FCR
	Ctrl	310	1147^b^	1874	1.63^a^	394^d^	694	1.76^a^	1541^c^	2568	1.67^a^
300ppm	T2(Sand)	315	1278^ab^	1889	1.48^ab^	410^d^	696	1.70^ab^	1688^b^	2585	1.53^b^
	T3 (Ash)	312	1276^ab^	1907	1.49^ab^	415^d^	709	1.71^ab^	1691^b^	2616	1.55^b^
	T4 (NSis)	309	1341^a^	1856	1.38^b^	464^c^	720	1.55^c^	1805^a^	2576	1.43^c^
	T5 (NSia)	308	1387^a^	1869	1.35^b^	472^c^	740	1.57^c^	1859^a^	2609	1.40^c^
600ppm	T6(Sand)	309	1286^ab^	1860	1.45^ab^	397^d^	683	1.72^ab^	1683^b^	2543	1.51^b^
	T7 (Ash)	310	1288^ab^	1889	1.47^ab^	408^d^	676	1.66^ab^	1696^b^	2565	1.52^b^
	T8 (NSis)	310	1368^a^	1818	1.33^b^	487^b^	687	1.41^d^	1855^a^	2505	1.35^c^
	T9 (NSia)	313	1406^a^	1843	1.31^b^	511^a^	723	1.42^d^	1917^a^	2566	1.34^c^
Significance		NS	**	NS	**	***	NS	***	***	NS	***
SE		± 0.84	± 19.18	± 7.77	± 0.02	± 8.23	± 5.72	± 0.02	± 25.64	± 10.22	± 0.02
Main effects											
Source											
CTRL		310	1147^c^	1874	1.63^a^	394^b^	694	1.76^a^	1541^c^	2568	1.67^a^
Sand		312	1282^b^	1875	1.47^b^	404^b^	690	1.71^a^	1686^b^	2564	1.52^b^
Ash		311	1282^b^	1898	1.48^b^	412^b^	693	1.69^a^	1694^b^	2591	1.54^b^
NSis		310	1355^a^	1837	1.36^c^	476^a^	704	1.48^b^	1830^a^	2541	1.39^c^
NSia		311	1397^a^	1856	1.33^c^	492^a^	732	1.50^b^	1888^a^	2588	1.37^c^
SE of means		± 4.81	± 23.82	± 14.76	± 0.03	± 10.70	± 12.82	± 0.02	± 31.64	± 23.45	± 0.02
Level ppm											
0 ppm		310	1147^b^	1874	1.63^a^	394	694	1.76^a^	1541^b^	2568	1.67^a^
300 ppm		311	1321^a^	1880	1.43^b^	440	716	1.63^b^	1761^a^	2597	1.48^b^
600 ppm		311	1337^a^	1853	1.39^b^	451	692	1.55^b^	1788^a^	2545	1.43^b^
SE of means			± 20.70	± 11.55	± 0.03	± 13.74	± 17.42	± 0.03	± 32.24	± 21.33	± 0.02
Significances											
Source of variation		± 3.88									
Source effect		NS	**	NS	**	*	NS	***	***	NS	***
Level effect		NS	***	NS	**	NS	NS	***	***	NS	***
Source × Level		NS	**	NS	**	***	NS	***	***	NS	***

*P ≤ 0.05, **p < 0.001,***P < 0.0001, and NS, not significant (*P* > 0.05) indicate that there is no statistically significant difference between means in the same column. Normality of the data was confirmed using the Shapiro–Wilk test (*p* > 0.05).

The starting weight of birds in all treatments was similar (*P* > 0.05), about 310 g.

In the growing period, weight gain (WG) and feed conversion ratio (FCR) were significantly improved (*P* ≤ 0.05) for birds fed diets containing nano silicon dioxide produced from sand (NSis) at 300 ppm (NSis300), 600 ppm (NSis600) or from rice straw’s ash (NSia) at 300 ppm (NSia300) or 600 ppm (NSia600) compared to the control.

At the finisher phase, the best (*P* ≤ 0.05) WG recorded for NSis600. The high level (600 ppm) was significantly (*P* ≤ 0.05) better than the low level (300 ppm) in WG and FCR for birds fed diets containing nano silicon dioxide produced from sand or rice straw’s ash. Addition of NSis or NSia at both levels significantly (*P* ≤ 0.05) improved WG and FCR compared to the control.

At the overall period, All treatments significantly (*P* ≤ 0.05) improved WG and FCR compared to the control group. Whereas, the best results for WG and FCR were for chicks fed diets enriched with nano silicon dioxide produced from sand or rice straw’s ash.

While, feed consumption did not differ significantly (*P* > 0.05) from the control group across all treatments, throughout the experiment.

The main source of impact: All sources led to improved growth and productive performance by enhancing BWG and reducing the FCR compared to the control in grower and overall periods, while the best results were recorded for nano silica irrespective of its source in all periods. No significant differences in feed intake were recorded among all sources and the control group.

The main level effect: adding two levels, 300 ppm or 600 ppm, improved BWG and FCR during the grower and overall periods, but only improved FCR during the finisher period. There were no significant differences in feed intake across all levels.

#### Blood proteins

**Fig. 2:**
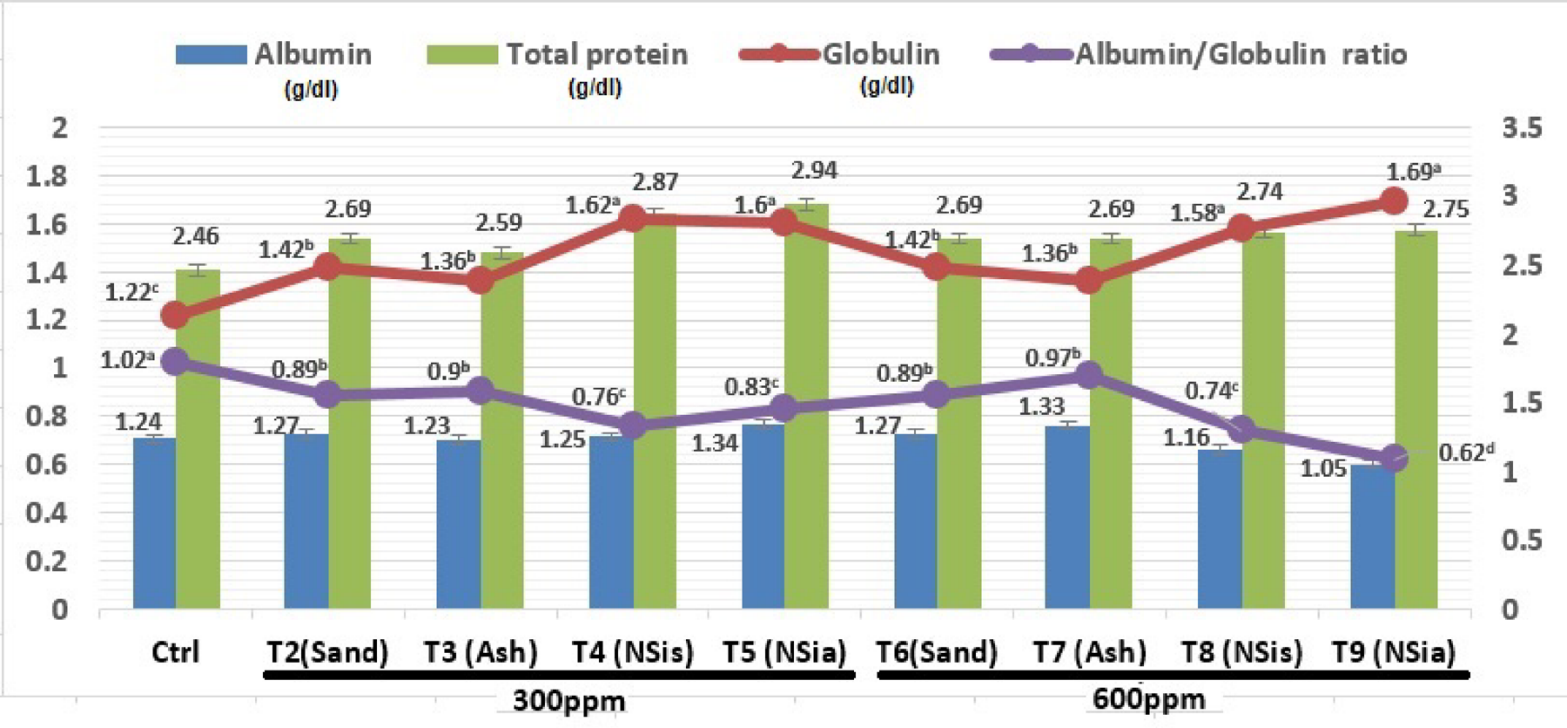
Influence of several dietary treatments on total protein, albumin, globulin and the albumin/globulin ratio.

Figure 2 shows the effects of several dietary treatments on total protein, albumin, globulin and albumin/globulin ratio.

There are no significant (*P* > 0.05) differences in total protein and albumin among all treatments. All treatments improved significantly (*P* ≤ 0.05) globulin and albumin/globulin ratio compared with control group. The best globulin and albumin/globulin ratio recorded for chicks fed diets enriched with nano silicon dioxide produced from sand or rice straw’s ash.

### Blood biochemical parameters

Table 3 shows the effects of several dietary treatments on biochemical blood parameters.

**Table 3:** The effects of several dietary treatments on biochemical blood parameters.

Item		Urea (g/dl)	ALP (IU/L)	ALT (U/L)	AST (U/L)
	Ctrl	2.66	146.4	13.30	202.7
300ppm	T2(Sand)	2.52	146.6	12.73	199.7
	T3 (Ash)	2.17	146.8	12.90	198
	T4 (NSis)	2.32	152	12.87	199.5
	T5 (NSia)	2.35	149.9	12.67	196.3
600ppm	T6(Sand)	2.10	151.7	12.87	195
	T7 (Ash)	2.20	148.2	13.27	198
	T8 (NSis)	2.01	148.6	13.17	194
	T9 (NSia)	2.04	148.4	13.23	196
Significance		NS	NS	NS	NS
SE		0.07	0.67	0.08	0.87

NS, not significant (*P* > 0.05) signifies that there is no statistically significant difference between means in the same column that are indicated with the same letter. Normality of the data was confirmed using the Shapiro–Wilk test (*p* > 0.05).

Urea, Alkaline phosphatase (ALP), aspartate aminotransferase (AST), and alanine aminotransferase (ALT) levels were not significantly different between the treatment groups and the control group (*P* > 0.05). As a result, it may be concluded that the experimental additives to the chick diets had no significant effect on bone, kidney, or liver functions.

### Antioxidants status

**Fig. 3:**
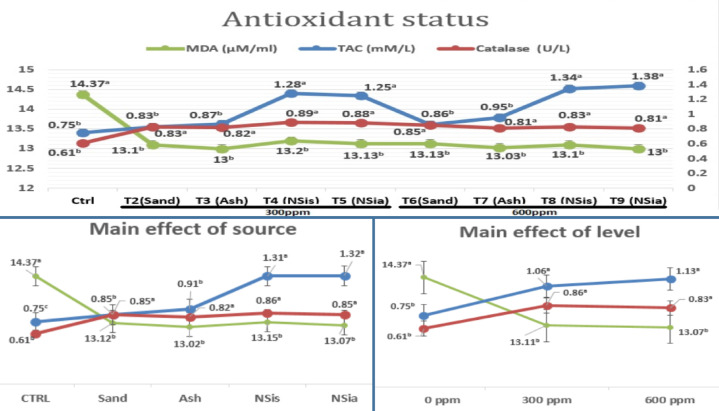
Influence of several dietary treatments on antioxidants status.

Figure 3 shows the effects of several dietary treatments on antioxidants status.

When nano silicon dioxide manufactured from sand or rice straw ash was added to the chicks’ diets, the total antioxidant capacity (TAC) increased significantly (*P* ≤ 0.05) compared with control. Furthermore, all treatments significantly (*P* ≤ 0.05) improved both catalase (CAT) and malondialdehyde (MDA) compared with the control.

The main effect of the source is that all the sources provided to the broiler showed better results for antioxidant status (with increases in TAC and CAT and a decrease in MDA) compared to the control. Additionally, nanosilica, regardless of where it came from, produced the best results in total antioxidant capacity. The main effect of the amount added improved antioxidant status at both levels (300 or 600 ppm) compared to the control, with no significant difference between the two levels. The main effect of level of addition led to improved antioxidant status under two levels (300 or 600 ppm) compared to the control without any significant difference between the two levels.

### Tibia bone characteristics

Table 4 shows the effects of different treatments on tibia bone characteristics.

**Table 4:** The effects of different treatments on tibia bone characteristics.

Item		Weight (g)	Length (cm)	Width (cm)
	Ctrl	9.52	8.53	0.66b
300ppm	T2(Sand)	9.27	8.24	0.67b
	T3 (Ash)	9.26	8.20	0.68b
	T4 (NSis)	10.54	8.76	0.74a
	T5 (NSia)	10.95	8.86	0.77a
600ppm	T6(Sand)	10.45	8.51	0.68b
	T7 (Ash)	10.21	8.53	0.75a
	T8 (NSis)	10.57	8.86	0.77a
	T9 (NSia)	11.10	8.90	0.78a
Significance		NS	NS	***
SE		0.19	0.06	0.01

*P ≤ 0.05, ***P < 0.0001, and NS, not significant (*P* > 0.05) indicate that there is no statistically significant difference between means in the same column. Normality of the data was confirmed using the Shapiro–Wilk test (*p* > 0.05).

No significant differences recorded for weight and length of tibia bone among all treatments. Whereas, birds fed diets enriched with NSis 300 ppm, NSia 300 ppm, Ash, NSis 600 ppm and NSia 600 ppm had the better tibia width compared with the control.

**Fig. 4:**
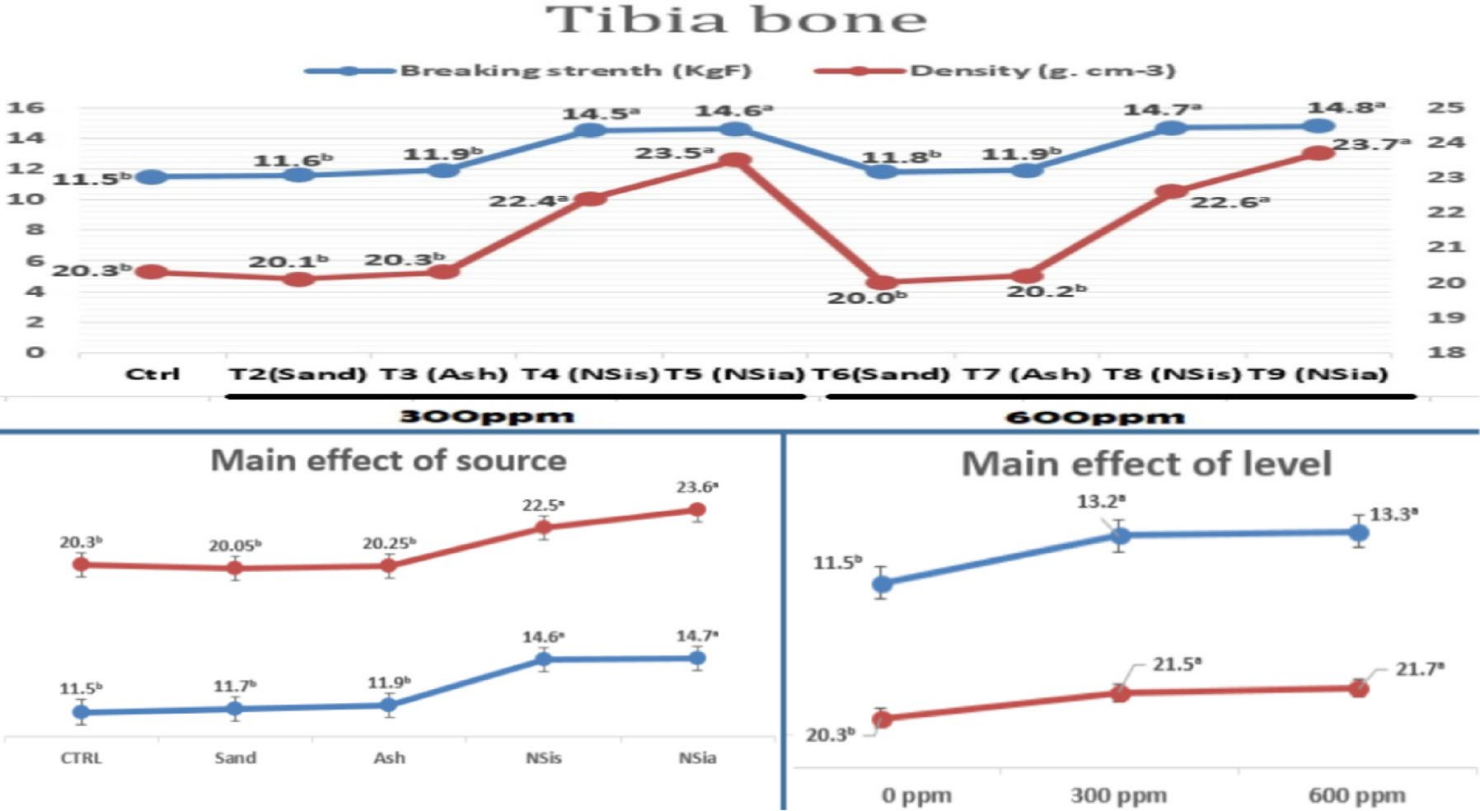
Influence of several dietary treatments on tibia bone mineral density and breaking strength.

Tibia bone mineral density (BMD) and breaking strength (BS) of chicks fed different experimental diets are shown in Fig. 4. Broiler chicks fed diets with nano silicon dioxide from different sources and levels , NSis_300_, NSia_300_, NSis_600_ and NSia_600_, gave the best values (*P* ≤ 0.05) in BMD and BS.

**Fig. 5:**
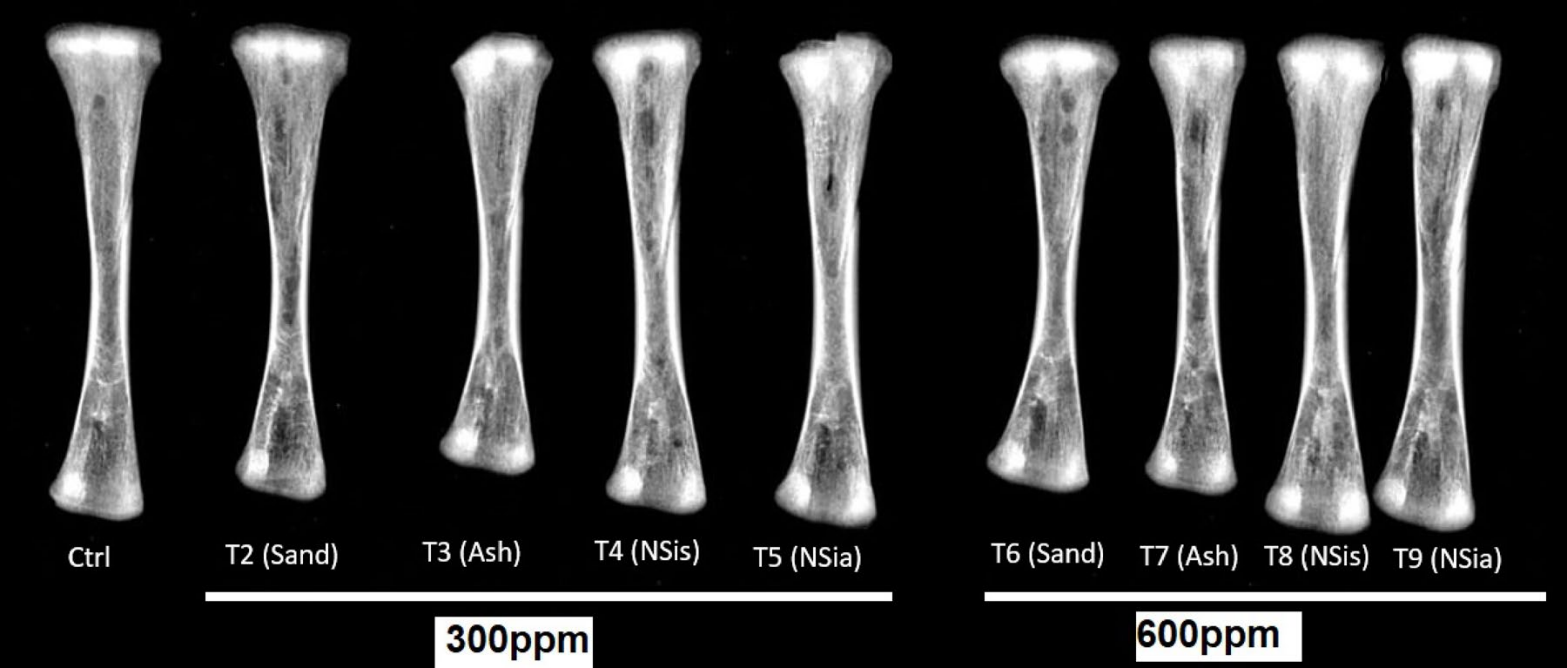
X-Ray image was taken on tibia bone.

**Fig. 6:**
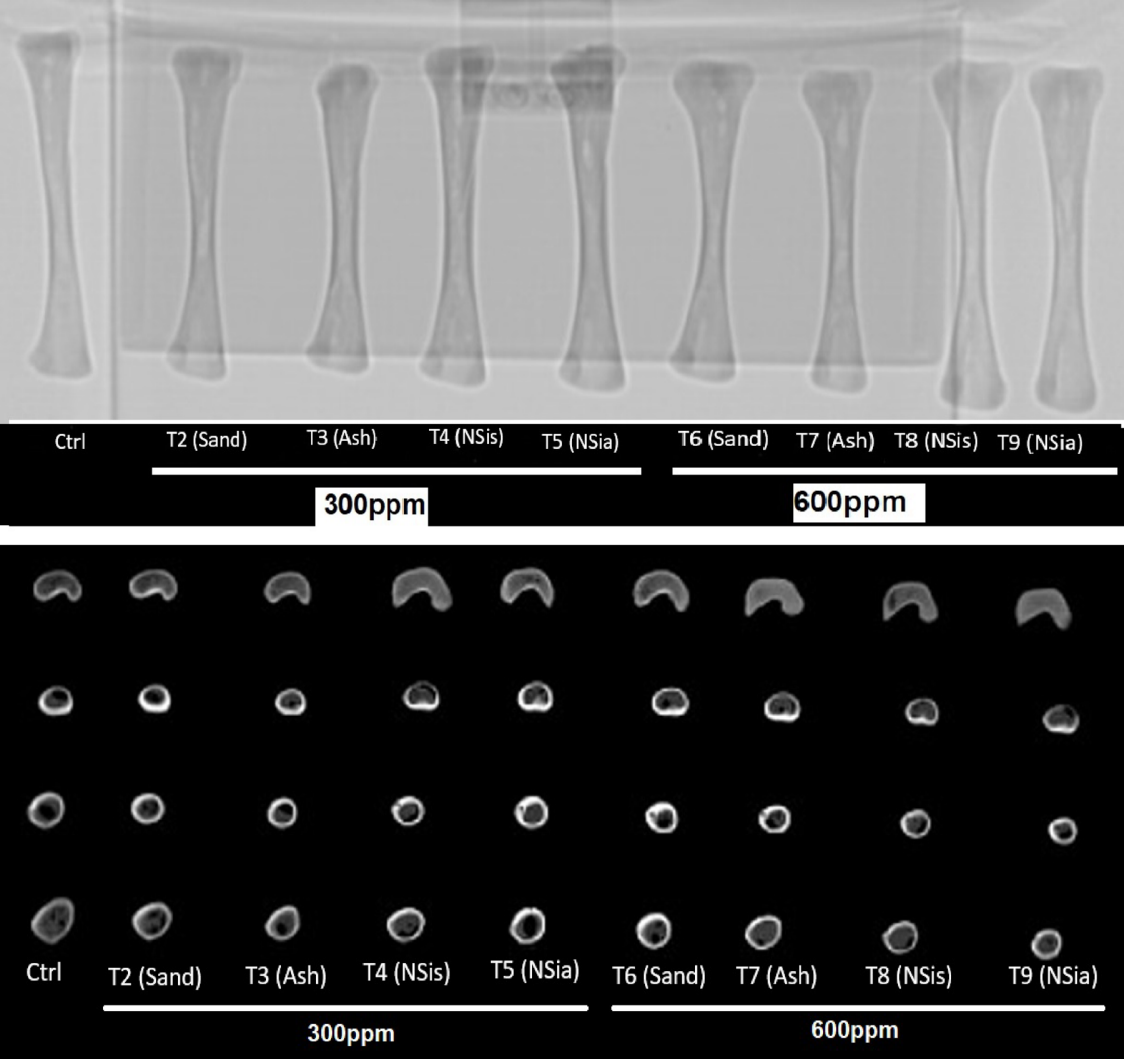
Radiographic image of the effect of different treatments on tibia bone scans using CT at 33 days of age.

Effect of different treatments at 33 days of age illustrated in radiographs in Fig. 5 and 6. The addition of the NSis and NSia at 300 and 600 ppm to the broiler diets significantly (*P* ≤ 0.05) enhanced BMD of tibia (Epiphysis and diaphysis) compared to the control.

The main effect of the source is that the incorporation of nano silica, irrespective of its origin—sand or rice straw ash—yields superior outcomes in BMD and BS compared to other treatments, with no significant differences seen between the two sources of nano silica. Also, the main impact of the level resulted in the addition of 300 or 600, demonstrating improvement in BMD and BS relative to the control group without any significant differences between two levels.

#### Thyroid hormones

**Fig. 7:**
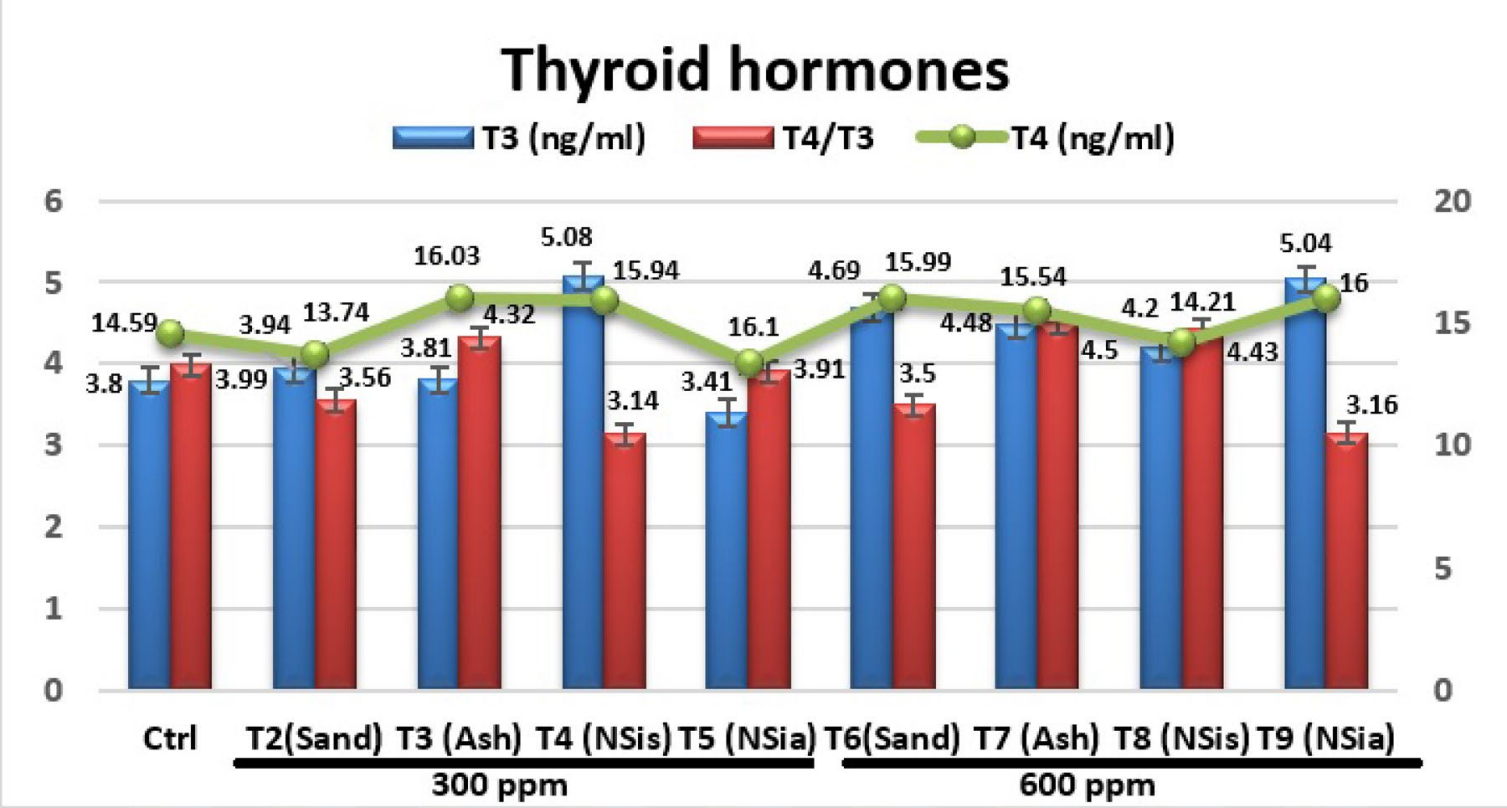
The effects of different treatments on thyroid hormones.

Figure 7 shows the effects of different treatments on thyroid hormones.

The levels of *Triiodothyronine* (T3), *Thyroxine* (T4) and the ratio between them were not affected significantly (*P* > 0.05) by the different treatments compared to the control group.

## Discussion

Addition of nano silicon dioxide produced from sand or rice straw’s ash at different levels gave the best (*P* ≤ 0.05) broiler productive performance, immune and antioxidants status and tibia bone characteristics without any negative effect (*P* > 0.05) on the thyroid hormones, bone, liver and kidney functions during 11–33 days of age compared to the control.

Smaller sizes (1–100 nm) of nanominerals make them more effective than their traditional counterparts while also improving their activity, reactivity, absorption, and utilization. So, this increases their bioavailability^4–6,12–14^. Nanominerals increase bioavailability in two ways: by protecting the gastrointestinal tract against chemical conditions and by improving transfer through the intestinal wall^15,16^.

Broiler body weight gain (BWG) and feed conversion ratio (FCR) at 35 days significantly improved when traditional ZnO was substituted with the nano form, with no adverse effects^26,27^. Moreover, adding 40 mg/kg ZnO NPs to broiler diets for five weeks resulted in significantly enhanced performance by improving both BWG and FCR compared to the control group^28,29^. In line with these findings, Samy et al.^30^ determined that broilers nourished with diets containing either green or chemically synthesized nano zinc exhibited superior BWG, and FCR relative to those fed traditional sources. Furthermore, Abd-Elsamee et al.^31^ demonstrated that ZnO NPs yielded optimal outcomes for carcass weight and humoral immunity without adversely affecting thyroid hormones, liver, or renal function. With respect to oxidative stress, broilers administered 40 mg ZnO NPs/kg showed a significant reduction in malondialdehyde (MDA) levels compared to those receiving 40 mg Zn lysine/kg or the control^32^. In terms of skeletal development, broilers given nanominerals substituted exhibited substantial enhancements in tibia bone characteristics and development compared to those receiving inorganic or organic minerals^5,30,33,34^_._

Modern broiler strains may necessitate the use of bioavailable silicon supplements in their feed, Adding silica (1000 ppm) to the broiler diet increased weight gain and bone strength without changing bone mineral content^34^. Furthermore, Scholey et al.^35^ demonstrated that increased absorption of minerals into serum and bone in birds fed the novel type of silica improved bone strength and quality, as well as alleviating the typical lameness seen in the broiler business. Whereas, silicon from silicon sand (1000 ppm) was not absorbed into the bloodstream as Si by broilers, but that silicon from bioavailable silicon (250, 500, 750 and 1000 ppm) did. The bioavailable silicon boosted significantly the strength of both tibia and femur at 21 days of age^1^. Supplementing broiler diets with silica (200 ppm) during the growing period from 22 to 35 days of age improved productive performance and economic efficiency (Enhancing both of Income over feed and chick cost and relative feed cost by 11.7 and 4.16%, respectively)^36^. Ghazalah et al.^37^ showed that the addition of nanosilica to broiler diets at 0.20% or bentonite at 0.50% enhanced broiler performance resulting from the addition of nanosilica at 0.20%. In addition. Burton et al.^18^ found that broiler meat and bone quality were improved when silicon was added to the diet as a mineral supplement. Nakhon et al.^38^ demonstrated that silicon can minimize lameness in broilers.

Faryadi and Sheikhahmadi^39^ found that supplementing the diet of laying quails with 4000 mg/kg nSiO2 improved performance, egg weight, egg shell weight, and density of bone by stimulating the gastrointestinal tract, resulting in an optimal supply of necessary nutrients. Also, Ratriyanto et al.^40^ revealed that supplementing quails with activated silicon dioxide and Betaine increased their growth performance from 7 to 42 days of age. Also, boosted quail productivity during the laying phase (35 to 70 days), as evidenced by increased egg production and egg weight. Silicon positively affects bone and mineral metabolism^2^. Adding silicon to broiler diets increases bone strength and density^41^.

## Mechanisms of silica NPs underlying the observed impacts

The observed enhancement in nutrient absorption and mineral bioavailability in broilers supplemented with silica nanoparticles (SiNPs) can be attributed to several interrelated mechanisms. Due to their ultrafine size and high surface area, SiNPs can interact more effectively with the intestinal mucosa, facilitating improved dissolution and transport of co-administered nutrients, such as calcium and phosphorus^42,43^. Their nanostructure allows them to penetrate the intestinal epithelium via transcellular pathways or through tight junction modulation, thereby increasing the permeability of the gut lining^44,45^.

A crucial mechanism involves the interaction of SiNPs with calcium metabolism. Where silica has been suggested to influence calcium homeostasis by modulating osteogenic signaling pathways or by altering intestinal calcium transport. In broilers, calcium absorption is a tightly regulated process involving calcium-binding proteins such as calbindin-D28k and transporters like TRPV6. Recent findings suggest that SiNPs may upregulate these calcium transport mechanisms either directly or via indirect signaling pathways. The nanoparticles could also modify the expression of genes involved in mineral metabolism, thereby enhancing the efficiency of calcium uptake and utilization for bone development and muscle growth^46,47^.

Furthermore, SiNPs may modulate the gut microbiota in a manner that supports better nutrient absorption. Certain bacterial communities stimulated by SiNPs could improve intestinal health and enzymatic activity, thereby creating a more favorable environment for nutrient uptake. Additionally, the surface charge and reactivity of SiNPs may aid in forming bioavailable complexes with dietary minerals, preventing their precipitation and loss in feces^48^.

These synergistic effects contribute to the improved performance parameters observed in broilers, such as higher body weight gain, better feed conversion ratio (FCR), and improved bone mineralization^5,22,49,50^.

## Conclusion

Silicon plays an important function in bone formation and quality in animals, so it is necessary to add it to poultry diets. According to our findings, Broiler diets supplemented with nano silicon dioxide derived from sand or rice straw’s ash could boost productivity, tibia bone development and quality and antioxidant status without any negative effects on kidney and liver functions.

## Data Availability

The datasets used and/or analyzed during the current study available from the corresponding author on reasonable request.
